# Effects of dietary probiotic (*Clostridium butyricum* I9, *C. butyricum* G15, or *Paraclostridium bifermentans* X13) on growth, digestive enzyme activities, immunity, and intestinal microbiota of Pacific white shrimp (*Penaeus vannamei*)

**DOI:** 10.3389/fmicb.2024.1479446

**Published:** 2024-11-27

**Authors:** Wei Yang, Huifen Liang, Ruhan Chen, Zhinuo Du, Taoqiu Deng, Yuqing Zheng, Ying Song, Yanchuang Duan, Junyuan Lin, Md. Akibul Hasan Bakky, Ngoc Tuan Tran, Ming Zhang, Shengkang Li

**Affiliations:** ^1^Guangdong Provincial Key Laboratory of Marine Biology, Shantou University, Shantou, China; ^2^Institute of Marine Sciences, Shantou University, Shantou, China

**Keywords:** *Penaeus vannamei*, probiotics, survival, *Vibrio parahaemolyticus* infection, shrimp

## Abstract

Pacific white shrimp (*Penaeus vannamei*) is one of the most productive and economically important species globally. However, the development and continuous expansion of the farming scale led to an increase in the risk of disease occurrence in shrimp farming. The application of probiotics as an effective method for controlling diseases in aquaculture has been widely considered. In shrimp farming, several probiotics have been used and shown benefits to the health of the host. To diverse the sources of bacterial species as probiotics in shrimp farming, in this study, we aimed to elucidate the effects of dietary probiotics (*Clostridium butyricum* I9 (I9), *Clostridium butyricum* G15 (G15), or *Paraclostridium bifermentans* X13) on the growth, immune response and intestinal microbiome of white shrimp. Shrimps were fed with diets containing either phosphate-buffered saline (PBS), I9 (10^7^ CFU/g feed), G15 (10^7^ CFU/g feed), or X13 (10^7^ CFU/g feed) for 30 days and followed by the challenge with *Vibrio parahaemolyticus* (*Vp*). The results showed that the survival rate, body weight gain, and special growth rate of shrimps in the I9, X13, and G15 groups significantly increased, compared to the PBS. The supplementation of probiotics increased the content of short-chain fatty acids and effectively maintained the normal morphology and structure of the intestinal tract and hepatopancreas. The I9, X13, or G15 groups showed a positive change in the diversity and abundance of gut bacteria. There was a significant up-regulation of CTL, SOD, proPO, Crustin, PEN2-4, and ALF1-3 genes in shrimps in the I9, X13, and G15. Additionally, dietary probiotics significantly increased the survival rate, maintained the intestinal structure, promoted the activities of SOD, AKP, ACP, and T-AOC enzymes, and reduced the level of MDA in shrimps after *Vp* infection. In conclusion, dietary supplementation of I9, G15, or X13 improved the growth, immunity, and disease resistance of Pacific white shrimp, providing a scientific basis for shrimp farming.

## 1 Introduction

Crustaceans are the second largest subphylum in the world. Pacific white shrimp (*Penaeus vannamei*) is a member of the phylum Arthropoda, class Crustacea, order Decapoda, and is one of the three most productive and economically important shrimp species globally (Cahú et al., 2012; Cornejo-Granados et al., 2017; Liu et al., 2017; Jin et al., 2018; Tran et al., 2023). With the rapid development of aquaculture, high-density culture leads to the risk of disease occurrence in intensive aquaculture, which has become one of the most important factors limiting the improvement of aquaculture production (Kumar et al., 2014). In shrimp aquaculture, diseases caused by bacteria (such as *Vibrio parahaemolyticus*-*Vp*) and viruses (white spot syndrome virus-WSSV) have negative effects on the development of shrimp aquaculture (Aziz et al., 2012; Chang et al., 2012; Nimrat et al., 2012). Antibiotics are frequently applied to minimize the adverse effects of diseases in shrimp farming. However, prolonged use can result in the emergence of antibiotic resistance, and cause water pollution in aquaculture systems (Mo et al., 2015; Okocha et al., 2018). Therefore, finding environmentally friendly farming methods is important in disease control and environmental protection in the shrimp aquaculture industry.

Previous studies proved that active ingredients from plants, chemicals, and probiotics as additives in cultured aquatic animals can improve health status and prevent diseases (Pérez-Sánchez et al., 2018). In recent years, probiotics have emerged as an antibiotic-free farming strategy to prevent and reduce the risk of diseases in aquaculture. The addition of *Bacillus tequilensis* AP BFT3 to the diets of Pacific white shrimp (*P. vannamei*) significantly upregulated the expression of Litv1, tropomyosin, and myosin genes, and improved the growth performance (Panigrahi et al., 2022). Dietary supplementation of *Rhodobacter sphaeroides* significantly promoted the growth performance and the relative abundance of beneficial microbiota (including *Paracoccus*, and *Sulfitobacter*) in the gut of Pacific white shrimp (Song et al., 2024). Another study has demonstrated that probiotics, *Lactobacillus reuteri* and *Pediococcus acidilactici*, can increase the body weight gain, specific growth rate, and survival rate, as well as, decrease the feed conversion rate of Pacific white shrimp after 8 weeks of feeding (Wu et al., 2022). The results of these studies showed that probiotics as feed additives have beneficial effects on improving health status and preventing diseases in shrimp farming.

In aquatic animals, the production of short-chain fatty acids (especially butyric acid) by butyrate-producing bacteria through the fermentation of carbohydrates promotes the regeneration and repair of the intestinal epithelium as well as the regulation of a healthy intestinal microecological environment (Duan et al., 2017; Tran et al., 2020a). Supplementing butyrate-producing bacteria into feeds can regulate the intestinal microbiota, inhibit the growth of pathogenic bacteria, and improve host immunity and host resistance to pathogenic bacteria (Tran et al., 2020b). Of the butyrate-producing bacteria, *Clostridium butyricum* is the most common bacterium used in aquaculture. The previous study reported that Pacific white shrimps fed a diet supplemented with *C. butyricum* for 42 days showed an increased body weight, growth rate, survival rate, and resistance to *Vp* infection (Li et al., 2019). It also found that the application of *C. butyricum* CG30 could stimulate the alkaline phosphatase (AKP), down-regulate the expression of phenol oxidase (proPO), α-2-macroglobulin (A2M), and anti-lipopolysaccharide factor (ALF), and increased the survival rate of Pacific white shrimp after infection with *V. alginolyticus* (Wang et al., 2023). Thus, *C. butyricum* is an excellent class of probiotics that have been shown to have important roles in aquatic animals such as regulating intestinal microecological balance and improving intestinal health (Liang et al., 2023).

To diversify the potential sources used in shrimp farming, in this study, we evaluated the effects of three bacteria, *C. butyricum* I9, *C. butyricum* G15, and *Paraclostridium bifermentans* X13 on the growth performance, gut microbiota, antioxidant capacity, immune response, and disease resistance in Pacific white shrimp. These bacterial strains, I9, G15, and X13, were previously isolated from the intestine of white seabass (*Atractoscion nobilis*) and were demonstrated to show a positive contribution to the growth and health of white seabass (unpublished data). The results of this study provide an important scientific basis for the potential application of these bacteria as probiotics in shrimp aquaculture.

## 2 Materials and methods

### 2.1 Bacteria strain, source of shrimp, culture conditions

The probiotic bacteria, I9, G15 and X13, used in this study were previously isolated from the gastrointestinal tract of white seabass and stored in the Mud crab Disease and Immunity Lab, Shantou University (Shantou, China). The bacteria were separately cultured in PY medium at 28°C and kept shaking at 120 rpm for 24 h (Liang et al., 2023). After 24 h of incubation, the bacterial solution was centrifuged at 6,000 rpm for 10 min to collect the bacterial cells, which were washed with sterile PBS three times and resuspended with sterile PBS buffer. The final biomass of the concentration was adjusted to 1 × 10^7^ CFU/mL.

Six hundred healthy juvenile shrimps (initial body weight: 8.1 ± 0.2 g; initial body length: 11.5 ± 0.4 cm) were cultured in the Laboratory Haikang Breeding Base of Fujian Dabeinong Huayou Aquatic Technology Group Co. Ltd. (Fujian, China) in a filtered aerated seawater system (water temperature: 28 ± 2°C, pH: 7.2 ± 0.4, dissolved oxygen: >6 mg/L, ammonia nitrogen: <0.2 mg/L, and nitrite nitrogen: <0.05 mg/L) for 5 days. The stocking density was 50 shrimps/tanks (300 L water). During the acclimatization period, the shrimps were fed with commercial pellet feed (≥43% crude protein, ≤15% crude ash, ≥5.0% crude lipid, ≤5.0% crude fiber, ≥2.6% lysine, ≤11% moisture, and 0.9–2.0% total phosphorus) purchased from Fujian Dabeinong Huayou Aquatic Technology Group Co., Ltd. (Fujian, China). The culture water was exchanged twice daily (each exchange rate was about 20%). The feed residues and feces were siphoned simultaneously during the water exchange time.

### 2.2 Feeding experiment

The shrimps were randomly divided into four groups: PBS (shrimps were fed basal diet supplemented with sterile PBS), I9 (shrimps were fed basal diet supplemented with I9), G15 (shrimps were fed basal diet supplemented with G15), and X13 (shrimps were fed basal diet supplemented with X13). The basal feed was directly sprayed with PBS or bacterial solution (1 × 10^7^ CFU/g feed), placed in an oven at 40°C for 30 min, and stored in a refrigerator at 4°C. Shrimps were fed twice daily (at 6:00 and 15:00) for 30 days. During the culture period, the water was exchanged twice daily, and feed residues and feces were cleaned up. The mortality of shrimp was recorded.

### 2.3 Growth parameters

The survival rate, weight gain, length gain, and specific growth rate of shrimp were estimated at the end of the culture experiment (30 days), and each growth parameter was calculated as follows:

Survival rate (SR,%) = (Final shrimp number)/(Initial shrimp number) × 100
Specific growth rate (SGR,%/day) = [(ln Final body weight – ln Initial body weight)/t] × 100%
Weight gain rate (WGR,%) = (Final body weight-Initial body weight)/(Initial body weight) × 100
Length gain (%) = [(Final body length - Initial body length)/Initial body length] × 100%

### 2.4 Sample collection and processing

At the end of the culture experiment, tissues including hemolymph, intestine, hepatopancreas, and intestinal contents of shrimp were collected, immediately placed in liquid nitrogen for rapid freezing, and then stored at −80°C for further use. The intestine and hepatopancreas tissues were collected and fixed with 4% paraformaldehyde for histopathological observation.

Hepatopancreas were collected and homogenized in sterile saline (with a ratio of 1:9, w/v). The supernatant (10%) was obtained by centrifugation at 2,500 rpm for 10 min and used for analyzing enzyme activities. The activities of enzymes, including superoxide dismutase (SOD), alanine transaminase (ALT), aminotransferase (AST), total antioxidant capacity (T-AOC), catalase (CAT), alkaline phosphatase (AKP), acid phosphatase (ACP), α-amylase (AMS), and malonaldehyde (MDA), were assayed using commercial kits (Nanjing Jiancheng Institute of Bioengineering, Nanjing, China) according to the manufacturer’s instructions.

The intestinal contents were mixed homogeneously with sterile saline in a ratio of 1:9 (w/v) and the mixture was centrifuged at 12,000 rpm for 10 min. The supernatant was collected, adjusted to pH 2-3 (using sulfuric acid), filtrated through a 0.22-μm filter membrane, and used for detecting SCFAs. The production of SCFAs was estimated using an Agilent GC6890N Network Gas Chromatograph (Agilent Technologies, Santa Clara, CA, USA), previously described by Liang et al. (2023).

### 2.5 RNA extraction and RT-qPCR

The intestines were washed with sterile PBS buffer to remove the contents, and the total RNA was extracted from the intestine using a commercial RNA extraction kit (Shanghai Feijie Biotechnology Co., Ltd. Shanghai, China) according to the manufacturer’s instructions. The quality and quantity of total RNA were evaluated and subsequently used as templates for cDNA synthesis using the reverse transcription kit (Tiangen, Beijing, China). The cDNA concentration was adjusted to 200 ng/μL and used for RT-qPCR.

The RT-qPCR reactions were performed in triplicate in a 20 μL mixture containing 1 μL (each of forward and reverse primers), 1 μL of cDNA, 7 μL of ddH_2_O, and 10 μL of 2 × PCR Mix. The reaction conditions include three steps: firstly, 95°C for 30 s, and then 95°C for 5 s for 40 cycles. Finally, 60°C for 20 s and melting curve analysis (65°C to 95°C). A Premix Ex Taq (Probe qPCR) (TaKaRa, Dalian, China) was used to analyze the expression of C-type lectin (CTL), superoxide dismutase (SOD), prophenoloxidase (proPO) and antimicrobial peptides (AMPs, including Crustin, penaeidin-PEN2-4 and anti-lipopolysaccharide factors-ALF1-3). β-actin gene was used as the reference gene. The relative expressions of these genes were normalized to β-actin gene expression and analyzed by the 2^–ΔΔCt^ algorithm.

### 2.6 DNA extraction, PCR amplification, and 16S rDNA sequencing

The intestinal contents were used for DNA extraction using the QIAamp Fast DNA Stool Mini Kit (Qiagen, Germany) following the manufacturer’s instructions. For each sample, DNA was extracted in triplicate and finally pooled together. The quality of the extracted DNA was ascertained by 1.0% agarose gel electrophoresis and the concentration was measured using a NanoDrop One (Thermo Scientific, USA). DNA samples were kept at −20°C until further analysis. The PCR products were sent to a commercial company (Novogenge Biotech Co., Ltd., Beijing, China) for sequencing.

The reads of each sample were spliced using FLASH (Version 1.2.11)^1^ (Magoè and Salzberg, 2011) to obtain raw Tags data (Raw Tags). The reverse primer sequences were matched and the remaining sequences were cut out using Cutadapt software. The Raw Tags were then filtered for high quality using fastp software (Version 0.23.1) to obtain Clean Tags (Bokulich et al., 2013). The Clean Tags were compared with the species annotation database and chimeric sequences were removed to obtain Effective Tags (Edgar et al., 2011). Effective Tags were clustered using the Uparse algorithm (Uparse v7.0.1001)^2^ (Edgar, 2013), the sequences were clustered with 97% consistency (Identity) into OTUs (Operational Taxonomic Unit), after which the OTUs were annotated to obtain species-specific classification. Finally, the data of each sample were homogenized and analyzed for alpha diversity and beta diversity based on OTUs.

To assess community richness and sample size, alpha diversity analysis (including Chao1, ACE, Shannon, and Simpson) was performed using the Qiime software (Version 1.9.1). Dilution curves were plotted using the R software (Version 4.0.3). To assess community diversity, beta diversity analysis based on weighted and unweighted distances was performed using Qiime software. Heatmaps showing unifrac distances between samples were plotted. Cluster trees were drawn for evolutionary classification using the UPGMA.tre function. Principal Component Analysis (PCA) and Principal Coordinate Analysis (PCoA) were performed using ade4 and ggplot in R software to reduce the dimensionality of the original variables and obtain and visualize the principal coordinates from the data. In order to reveal the differentiation of community structure, LEfSe was analyzed and plotted using vegan and ggplot2 in R software.

### 2.7 *Vibrio parahaemolyticus* infection experiment

At the end of the feeding experiment, the shrimps in groups were divided into PBS, PBS + *Vp* (*Vp*), I9 + *Vp*, X13 + *Vp*, and G15 + *Vp*. A volume of sterile PBS or 200 μL of *Vp* (1 × 10^6^ CFU/mL) was injected into the corresponding experimental groups. The culture conditions were kept consistent with the culture experiment, and the survival rate of shrimp was recorded 3 days after infection with *Vp*. At the end of the infection, tissues (intestinal content, intestine and hepatopancreas) were collected from shrimps in each group. Intestinal content was used for SCFA detection. The intestines and hepatopancreas were kept in 4% polymethanol tissue fixative to observe tissue morphology and structure by H&E staining. The hepatopancreas was homogenized in sterile saline (with a ratio of 1:9, w/v). The supernatant (10%) obtained by centrifugation at 2,500 rpm for 10 min was used for enzyme activity analysis. The activities of enzymes, including SOD, ALT, AST, T-AOC, CAT, AKP, ACP, AMS, and MDA were assayed using kits manufactured by Nanjing Jiancheng Institute of Bioengineering (Nanjing, China).

### 2.8 Statistical analysis

The data in the results were set in three replications. All data were expressed as mean ± S.D. The comparative analysis of variations among distinct experimental groups was conducted utilizing ANOVA, followed by Tukey’s HSD test. Asterisks are used to indicate the presence of statistically significant differences (*P < 0.05; **P < 0.01).

## 3 Results

### 3.1 Growth performance

**FIGURE 1:**
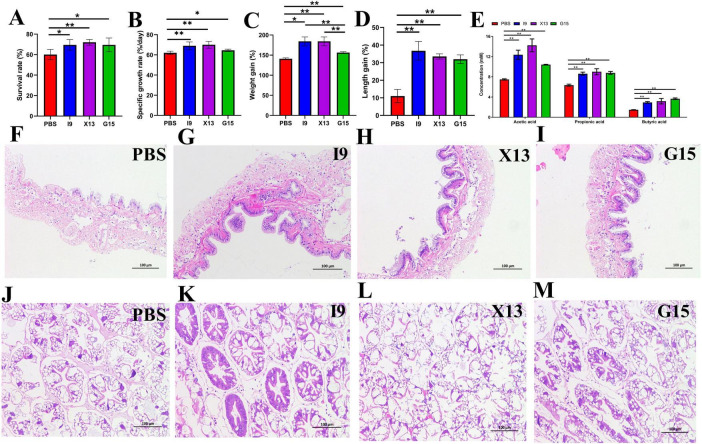
Growth performance of shrimp in different experimental groups. **(A–D)** Changes in survival rate, specific growth rate, weight growth rate, and length growth rate of shrimp. **(E)** Changes in short-chain fatty acids (SCFAs) content in the intestine. **(F–I)** Intestinal structure. **(J–M)** Hepatopancreas structure. The comparative analysis of variations among distinct experimental groups was conducted utilizing ANOVA, followed by Tukey’s HSD test. Asterisks indicate the presence of statistically significant differences (*P < 0.05; **P < 0.01).

The growth performance of shrimps fed with diets supplemented with I9, G15, or X13 was shown in Figure 1. The results showed that the addition of I9, G15, and X13 significantly increased the survival rate of shrimps by 69%, 69.2%, and 72% (*P* < 0.05), respectively, compared to the PBS (62.50%) (Figure 1A). The specific rate of shrimps in the I9 (69.04%), X13 (70.01%), and G15 (64.49%) was significantly increased as compared to PBS (62.02%) (Figure 1B). Moreover, the weight gain rate of shrimps in the I9 (185.51%), X13 (184.31%), and G15 (156.17%) was significantly increased as compared to PBS (145.85%) (Figure 1C). The length gain of shrimps in the I9 (36.76%), X13 (33.56%), and G15 (31.96%) significantly higher than the PBS (11.01%) (Figure 1D).

### 3.2 SCFA detection and intestinal structure observation

The results of the gas chromatographic analysis showed that the addition of probiotics significantly increased the content of acetic acid (I9: 12.37%, G15: 10.41%, and X13: 14.23%), propionic acid (I9: 8.61%, G15: 8.78%, and X13: 9.01%) and butyric acid (I9: 2.93%, G15: 3.17%, and X13: 3.66%). Among them, shrimp fed with X13 showed the highest total amount of SCFAs (26.41%), followed by I9 (23.92%) and G15 (22.85%) (Figure 1E).

Histological analysis revealed that intestinal villi were longer in the shrimps fed probiotic diets than in shrimps in the PBS (Figures 1F–I). The hepatopancreas had no vacuoles; the hepatic microsomes were more tightly arranged in the I9, G15, and X13 when compared with the PBS (Figures 1J–M). The results showed that the probiotic additions effectively maintain the healthy morphology of intestinal tissues and hepatopancreas.

### 3.3 Enzyme activity and immune response

**FIGURE 2:**
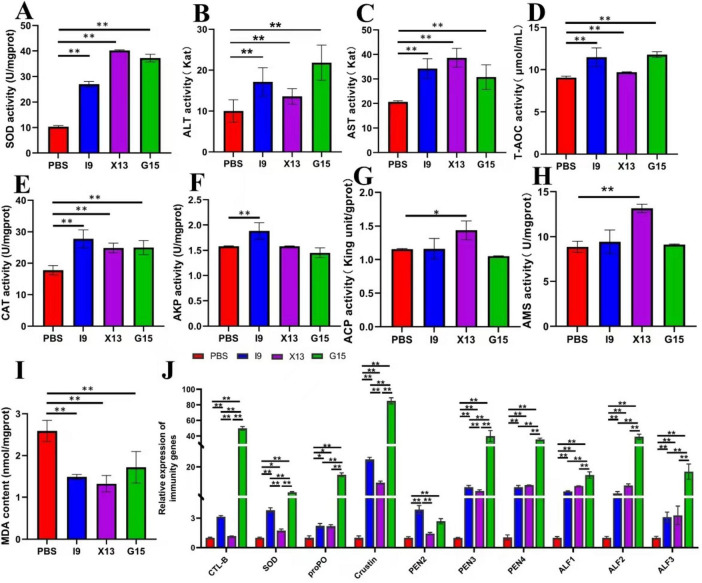
Changes in the immune function of shrimp in different experimental groups. **(A–I)** Represent the activities of enzymes (SOD, ALT, AST, TAOC, CAT, AKP, ACP, AMS, and MDA) in the hepatopancreas of different experimental groups. **(J)** Expression of immune-related genes (CTL, SOD, proPO, Crustin, PEN2-4, and ALF1-3) in the intestinal tissue. The comparative analysis of variations among distinct experimental groups was conducted utilizing ANOVA, followed by Tukey’s HSD test. Asterisks indicate the presence of statistically significant differences (*P < 0.05; **P < 0.01).

After the feeding experiment, the hepatopancreas of shrimp was used to examine the activities of SOD, ALT, AST, T-AOC, CAT, AKP, ACP, AMS, and MDA. Results showed that the addition of probiotics significantly enhanced the activities of enzymes, including SOD, ALT, AST, T-AOC, CAT, ACP, and AMS, but significantly decreased the content of MDA (*P* < 0.05) (Figures 2A–I). AKP activity was significantly higher in the I9, while ACP and AMS activities were significantly higher in the X13 than in the PBS (*P* < 0.05) (Figures 2A–I). These results suggested that probiotics could improve the antioxidant capacity of shrimps.

RT-qPCR was used to assess the expression of immune-related genes in the gut of shrimps after feeding with probiotics. The results showed that the genes CTL, SOD, proPO, Crustin, PEN2, PEN3, PEN4, ALF1, ALF2, and ALF3 were significantly up-regulated in the I9, X13, and G15 as compared with the PBS (*P* < 0.05). Among these genes, except for PEN2, which had the highest expression in the I9, the remaining genes had the highest expression in the G15 (Figure 2J). These results indicated that probiotic supplementation significantly improved the immune response of shrimps.

### 3.4 Changes in intestinal microbiota

**FIGURE 3:**
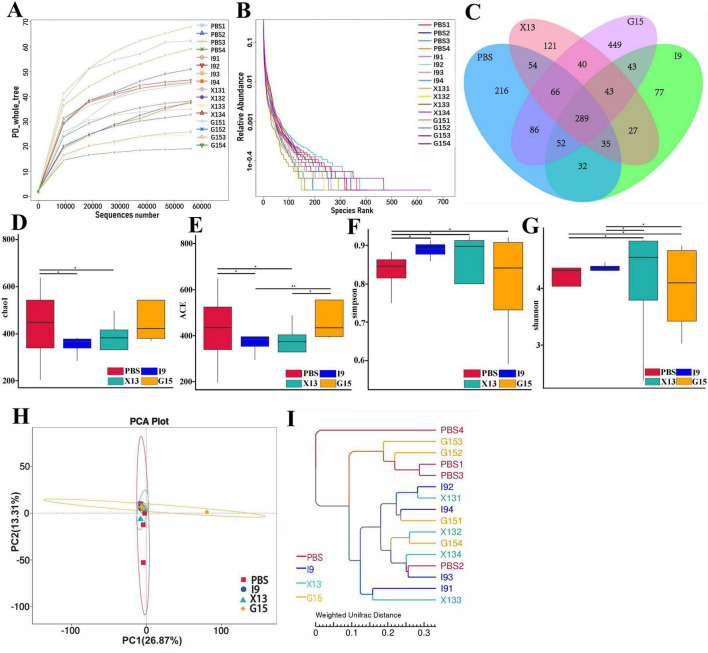
Changes in the diversity of gut microbiota in different experimental groups. **(A,B)** Sparsity curves showing the abundance of gut microbiota in each group. **(C)** Number of shared and unique OTUs between groups. **(D–G)** Diversity and richness of the gut microbiota (based on Chao1, ACE, Simpson, and Shannon indices). **(H)** Community structure similarity between groups was expressed using the PCoA method based on weighted UniFrac distances. **(I)** The unweighted pairwise group method of average chained (UPGMA) clustering tree structure was used to represent the distribution of clusters among samples. The comparative analysis of variations among distinct experimental groups was conducted utilizing ANOVA, followed by Tukey’s HSD test. Asterisks indicate the presence of statistically significant differences (*P < 0.05; **P < 0.01).

Changes in the intestinal microbiota of shrimps after feeding with the diets supplemented with PBS, I9, G15, or X13 for 1 month were investigated using 16S rRNA sequencing analysis. Saturation and sparsity curves indicate sample saturation based on high-throughput sequencing analysis (Figure 3A). Good coverage was greater than 99% for all samples, indicating that the bacterial community in each sample was largely defined to the saturation depth for sequencing. Rank abundance curves distinguish abundance between samples (Figure 3B). A total of 1,593,224 high-quality reads were obtained from all samples and the raw data were uploaded to Genbank, with an accession number of PRJNA1143973. The reads were ranged from 64,477 to 117,644 (in PBS), 102,586 to 105,085 (in I9), 102,265 to 106,169 (in G15), and 70,636 to 10,5421 (in X13). There were 289 shared operational taxonomic units (OTUs) among groups and 216, 77, 449, and 121 unique OTUs were found in the PBS, I9, G15, and X13, respectively (Figure 3C).

For the alpha diversity, results showed that the Shannon and Simpson indices in the I9, G15, and X13 were significantly higher than in the PBS. In contrast, compared to the PBS, the ACE and Chao1 indices had a reduction in the I9 and G13 (Figures 3D–G). Beta diversity analysis revealed differences in bacterial communities in the PBS compared to other groups. The PCoA and WPGMA analyses showed that the bacterial community in the PBS was separated from others (Figures 3H, I).

**FIGURE 4:**
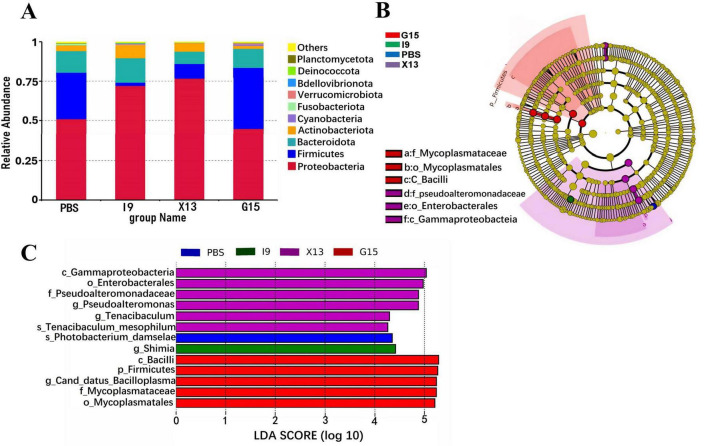
Composition of the gut microbial community in different experimental groups. **(A)** Relative abundance of gut microbiota at the phylum level. **(B)** LEfSe analysis of the phylogenetic distribution of microbial taxa. **(C)** Histogram of the distribution of LDA values indicating significant abundance of microbiota (LDA > 3.0).

Changes in the composition of the intestinal microbiota of shrimps fed different diets were investigated. The abundance of the top 10 bacterial phyla in groups is shown in Figure 4A. The increase in the relative abundance of Proteobacteria, Bacteroidota, Actinobacteriota, and Verrucomicrobiota (in I9), Firmicutes, Cyanobacteria, and Verrucomicrobiota (in G15), as well as, Proteobacteria and Actinobacteriota (in X13), as compared to the PBS (*P* < 0.05). Simultaneously, the relative abundance of Firmicutes and Fusobacteriota (in I9), Firmicutes, Bacteroidota, and Fusobacteriota (in X13), as well as, Actinobacteriota and Fusobacteriota (in G15) showed a decreasing tendency compared to the PBS.

The LEfSe (LDA > 3.0) was used to examine the involvement of specific bacterial taxa in different groups (Figures 4B, C). The bacterial taxa Gammaproteobacteria and Enterobacterales were significantly enriched in the X13, while the Firmicutes, Bacilli, Canddatus Bacilloplasma, Mycoplasmataceae and Mycoplasmateles were in the G15, as compared to the PBS.

**FIGURE 5:**
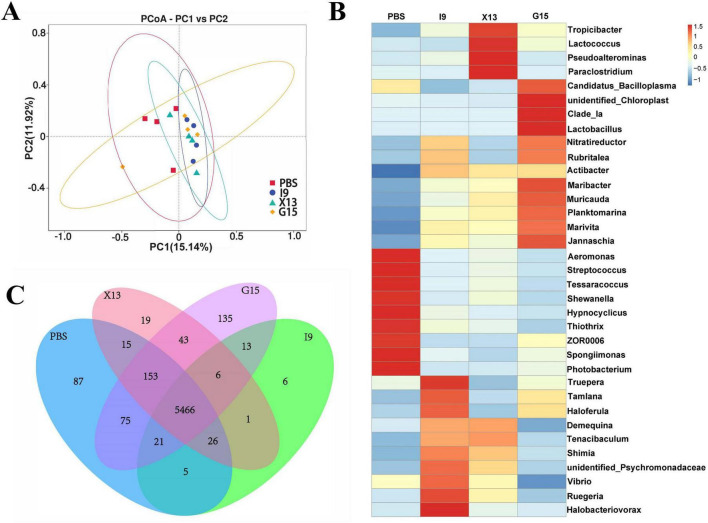
Functional profile of gut microbiota in different experimental groups. **(A)** Prediction of gut microbial function by PICRUSt. PCA downscaling plot showing the function of gut microbiota. **(B)** The number of genes between groups is presented by a Venn diagram. **(C)** Relative abundance and signaling pathways of the gut microbiota in the experimental groups were analyzed (at level 2).

The functional profile of intestinal microbiota in shrimps in the PBS, I9, G15, and X13 were obtained using PICRUSt analysis. The results of PCA showed a separation in microbial functions in the G15 compared to the PBS (Figure 5A). The distribution of shared and unique genes in different experimental groups is shown in Venn diagrams (Figure 5B). Figure 5B shows a total of 5,466 shared genes in all groups and 87, 6, 135, and 19 unique genes in the PBS, I9, G15, and X13, respectively. The differences in functions of microbiota among the groups were analyzed and are shown in Figure 5C. The supplementation of I9 and X13 significantly enhanced the pathways related to metabolism (including lipid and xenobiotics biodegradation, carbohydrates, terpenoids and polyketides, and biosynthesis), circulatory system, membrane system, and nervous system. The addition of G15 increased the cell growth and dead, energy metabolism and translation, but decreased the cardiovascular and respiratory functions, translation, and cardiovascular diseases.

### 3.5 Survival rate and immune response of shrimp after infection with *Vp*

**FIGURE 6:**
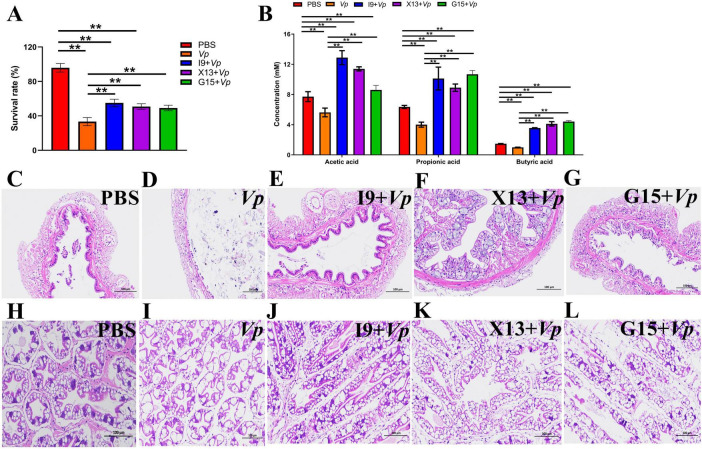
Changes in the survival, SCFAs production, and tissue histology of shrimps in different experimental groups after infection with *Vp*. **(A)** Survival rate. **(B)** Production of SCFAs in gut contents. **(C–G)** Intestinal structure. **(H–L)** Hepatopancreas structure. The comparative analysis of variations among distinct experimental groups was conducted utilizing ANOVA, followed by Tukey’s HSD test. Asterisks indicate the presence of statistically significant differences (*P < 0.05; **P < 0.01).

At the end of the feeding trial, shrimp in each group were subjected to *Vp* infection to evaluate the role of probiotic bacteria in improving the shrimp resistance to *Vp* infection. The results showed that, after *Vp* stimulation, the survival rate of shrimps in the I9 + *Vp* (55.00%), G15 + *Vp* (50.83%), and X13 + *Vp* (49.17%) was significantly higher than the positive control (*Vp*, 33.33%) (Figure 6A). Furthermore, our study found that the content of SCFAs (acetic, propionic, and butyric acids) was significantly higher in the I9 + *Vp* (26.53%), G15 + *Vp* (23.69%), and X13 + *Vp* (24.40%) as compared to the positive control (*Vp*, 10.66%) (Figure 6B). Histopathologically, the intestinal tissues of shrimp in the *Vp* group showed a reduced number and length of intestinal villi (Figures 6C–G). The hepatopancreatic tissues of the *Vp* group showed a lot of vacuolization, while the intestinal and hepatopancreatic tissues of shrimp in the I9 + *Vp*, G15 + *Vp*, and X13 + *Vp* showed a normal histological structure (Figures 6H–L).

**FIGURE 7:**
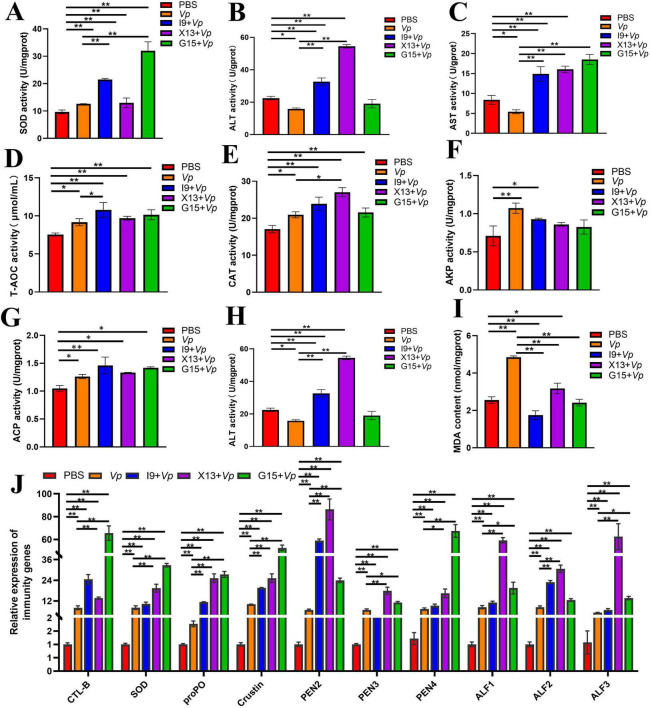
Changes in immune function in shrimps in different experimental groups after *Vp* infection. **(A–I)** Activities of enzymes (SOD, ALT, AST, T-AOC, CAT, AKP, ACP, AMS, and MDA) in the hepatopancreas. **(J)** Expression of immune-related genes (CTL, SOD, proPO, Crustin, PEN2-4, and ALF1-3) in the intestinal tissue. The comparative analysis of variations among distinct experimental groups was conducted utilizing ANOVA, followed by Tukey’s HSD test. Asterisks indicate the presence of statistically significant differences (*P < 0.05; **P < 0.01).

The activities of enzymes, including SOD, ALT, AST, T-AOC, CAT, AKP, and ACP, were significantly increased, but that of AMS and MDA were significantly decreased in the I9 + *Vp*, G15 + *Vp*, and X13 + *Vp* as compared to the positive control (*Vp*) (Figures 7A–I). In addition, the expression of immune-related genes, including CTL, SOD, proPO, Crustin, PEN2-4, and ALF1-3, were significantly increased in the I9 + *Vp*, G15 + *Vp*, and X13 + *Vp* after *Vp* infection, as compared to the PBS (Figure 7J). These results indicated that probiotic supplementation could improve the immunity and disease resistance of shrimps against *Vp* infection.

## 4 Discussion

The homeostasis in the gut is one of the most important factors affecting the growth and immunity of shrimps (Hou et al., 2018). Studies have shown that *Vp* and WSSV are the main factors that destroy microbial homeostasis and lead to mass mortality of shrimps in intensive aquaculture (Zhang et al., 2016). In shrimp culture, there are many ways to deal with pathogenic bacteria invasions, such as immune regulation, antibiotic treatment, good water quality management, reasonable feed management, genetic breeding, and regular monitoring and prevention. Among them, probiotics to protect the host from infection with bacterial pathogens have become a new way to prevent shrimp diseases in recent years (Xia et al., 2014). It has been reported that the addition of probiotics to feed not only promotes shrimp growth but also improves their antioxidant capacity and immunity by modulating the abundance of intestinal microbiota (Imaizumi et al., 2021).

In this study, feeding I9, G15, or X13 significantly increased the survival rate, specific growth rate, weight gain rate, and length gain rate of shrimps. A previous study reported that adding *C. butyricum* CB1-3 to diets can confer health benefits and modulate the gut microbiota of juvenile Pacific white shrimp under farming conditions (Zhang et al., 2023). Feeding *C. butyricum* CB for 56 days increased the total antioxidant capacity (T-AOC) content, lysozyme (LSZ) activity, and relative expression of Toll and immunodeficiency (Imd) genes, enhanced the growth performance of *L. vannamei* (Duan et al., 2017), which showed similar effects to the findings of this study. Herein, the production of SCFAs, such as acetic, propionic and butyric acids, in the shrimp’s intestine also increased significantly after feeding probiotics, which was similar to a previous report that human-origin probiotic *Lactobacilli paracasei* D3-5 and *Enterococci raffinosus* D24-1 can increase the content of SCFAs by modulating the gut microbiota in humans and mice, thereby ameliorating the dysbiosis of the gut microbiota (Nagpal et al., 2018). These results suggested that probiotic supplementation can significantly increase the production of SCFAs in the host intestine, effectively improving shrimp growth. In this study, feeding probiotics I9, G15, or X13 was effective in maintaining normal gut structure, as evidenced mainly by the number of visible intestinal villi and distinct folds. This is similar to the study that feeding *C. butyricum* CBG01 for 42 days significantly increased the intestinal villi height and the intestinal wall thickness in Pacific white shrimp (Li et al., 2019).

Moreover, the diets supplemented with I9, G15, or X13 promoted the activities of SOD, ALT, AST, T-AOC, CAT, ACP, and AMS, but decreased the MDA in the hepatopancreas of shrimps. This indicated that probiotic supplementation affects enzyme activity and improves immune performance, further promoting host growth. This agrees with the results of a previous study that the addition of *C. butyricum* G13 resulted in a significant increase in the activities of SOD, CAT, AKP, and ACP in mud crabs (Liang et al., 2023). Meanwhile, the up-regulation of immune-related genes in shrimps fed probiotic-supplemented diets herein indicated that these bacteria have beneficial effects on the immune system of shrimps. Our findings are supported by observations in a previous study that the expression of CAT, LYS, proPO, and SOD significantly increased in the hepatopancreas of mud crabs fed *Pediococcus pentosaceus* G11 (Yang et al., 2019). Dietary supplementation of *Bacillus subtilis* DCU or *Bacillus shortus* BP significantly stimulated the expression of CAT and proPO, thereby further promoting the immune performance and growth status of mud crabs (Wu et al., 2014). Thus, the results in this study indicated the positive effects of I9, G15, and X13 in stimulating the antioxidant capacity and immune response of shrimps.

The balance of intestinal microbiota has an important influence on the growth, development, digestion and absorption of nutrients, and immune function of animals (MacDonald et al., 2011). Intestinal homeostasis is associated with the inflammatory response, which influences the prevention of pathogen colonization (Maloy and Powrie, 2011). This study showed that the intestinal microbiota is closely related to body homeostasis; pathogens directly damage the intestinal barrier function, leading to intestinal microbiota dysbiosis. Xie et al. (2019) pointed out that dietary supplementation of 1,000 or 2,000 mg/kg probiotics significantly increased the richness of gut microbial diversity. This study found that the Chao1, ACE, Shannon, and Simpson indices were increased in the I9, G15, and X13 and that the microbial community in these groups (especially G15) was distinct from the PBS. The results indicated that the supplementation of probiotic bacteria effectively improved the diversity of gut microbiota, thus possibly maintaining immunity in shrimps.

The addition of I9, G15, and X13 showed significant differences in the composition of gut microbiota compared to the PBS, with a significant increase in the abundance of Proteobacteria and Firmicutes, suggesting that probiotics modulate the composition of intestinal microbiota. It is similar to the previous study that the application of *C. butyricum* changes the structure of gut microbiota, with an increase in the relative abundance of Proteobacteria and Firmicutes, in largemouth bass (*Micropterus salmoides*) (Meng et al., 2023). Another study showed that the abundance of Bacteroidetes and Firmicutes was increased by feeding the tilapia with the dietary addition of *C. butyricum* CB2. Feed supplementation with *C. butyricum* C2 promoted the growth of *Bifidobacterium bifidum*, but inhibited that of *Escherichia coli* and *Puccinia* in the midgut and hindgut of miiuy croaker (*Miichthys miiuy*) (Song and Wu, 2007). Furthermore, in the present study, the supplementation of I9 and X13 significantly enhanced the metabolic processes of lipid and xenobiotics biodegradation, amino carbohydrates and terpenoids. This is similar to the results of a previous study, in which dietary supplementation of *C. butyricum* increased the intestinal levels of lysophosphatidylcholine, and decreased the associated purine metabolism in Chinese mitten crabs (*Eriocheir sinensis*) (Gao et al., 2023). Collectively, probiotic supplementation stimulated the growth of beneficial bacteria and further influenced the growth and immune response of shrimp.

*Vp* infection is one of the infectious diseases in shrimp culture (Proespraiwong et al., 2023). The effects of I9, G15, or X13 on changes in the physiological and immune responses of shrimp after *Vp* infection were investigated. The results showed that the survival rate of shrimps was significantly improved after *Vp* infection in the probiotic-added groups. This is similar to the previous study that *C. butyricum* CB30 significantly improved the ability to survive and maintain normal gut structure of Pacific white shrimp after *V. alginolyticus* infection (Wang et al., 2023). In addition, the addition of phages (A3S and Vpms1) reduced the mortality of Pacific white shrimp infected with *Vp* (Lomelí-Ortega and Martínez-Díaz, 2014). In this current study, shrimp intestinal villi showed significant shedding and separation after *Vp* infection, but it was maintained as on the normal structure by feeding I9, G15, or X13. This is similar to the results of a previous study, feeding *C. butyricum* G13 maintained the normal intestinal structure of mud crabs after *Vp* infection (Liang et al., 2023). Furthermore, the addition of *Alcaligenes* sp. AFG22 showed a significant increase in the length, width, and area of the villi, which is beneficial for maintaining intestinal microbiota and promoting nutrient utilization in juvenile *Tor tambroides* (Asaduzzaman et al., 2018). The production of SCFAs, especially butyric acid, was significantly higher in the I9 + *Vp*, G15 + *Vp*, and X13 + *Vp* than in the *Vp* group. This is consistent with the findings in a previous study that *C. butyricum* CB1-3 can increase growth performance, digestibility, SCFAs in the intestine, immune response, and resistibility to ammonia stress in shrimp (*L. vannamei*) (Duan et al., 2017). Besides, the increase in the activities of enzymes (including SOD, ALT, AST, T-AOC, CAT, AKP and ACP) and a decrease in the AMS and MDA were found in the I9 + *Vp*, G15 + *Vp*, and X13 + *Vp*. This agrees with a previous study in *L. vannamei*, the dietary supplementation of *Enterobacter cloacae* E3 and *Lactobacillus lactis* L3 increased the activity of SOD, PPO, ACP, POD, CAT, and LZM and improved the immune performance of shrimp (Zuo et al., 2019). It is known that T-AOC, T-SOD, and CAT are important antioxidant enzymes that form the first layer of defense by preventing free radical formation (Wołonciej et al., 2016). AKP is a key component in defense against pathogens and oxidative stress (Callewaert and Michiels, 2010). The increased activity of these enzymes contributed to the antioxidant capacity and immunity of Pacific white shrimps in response to *Vp* infection. In this study, we also found that the expression of immune-related genes (including CTL, SOD, proPO, Crustin, and PEN4) was significantly increased in the I9 + *Vp*, G15 + *Vp*, and X13 + *Vp*. This indicated that I9, G15, and X13 stimulate the immune response to maintain gut health and protect the host against infection. The findings were similar to the case of Pacific white shrimp fed a diet added to *Aspergillus niger* (Zhang et al., 2023). Therefore, the results of this study demonstrated the positive effects of I9, G15, and X13 on the growth, gut microbiota, immune response, and disease resistance of Pacific white shrimp.

## 5 Conclusion

This study demonstrated that the bacteria I9, G15, and X13 have probiotic properties in Pacific white shrimp culture. The supplementation of the bacteria can effectively promote the growth, change the diversity and structure of the intestinal microbiota, with an increase in the abundance of the beneficial microbiota and the production of SCFAs in the intestine, maintain the normal intestinal structure, and enhance the antioxidant activity, immune response and disease resistance (against *Vp*) in shrimps. Taken together, our findings indicate that I9, G15, and X13 can be used as feed additives to promote growth and prevention of disease in shrimp culture.

## Data Availability

The datasets presented in this study can be found in online repositories. The names of the repository/repositories and accession number(s) can be found in this article/supplementary material.

## Footnotes

1
http://ccb.jhu.edu/software/FLASH/

2
http://www.drive5.com/uparse/
